# 2D and 3D numerical models to evaluate trabecular bone damage

**DOI:** 10.1007/s11517-021-02422-x

**Published:** 2021-09-01

**Authors:** Federica Buccino, Chiara Colombo, Daniel Hernando Lozano Duarte, Luca Rinaudo, Fabio Massimo Ulivieri, Laura Maria Vergani

**Affiliations:** 1 Department of Mechanical Engineering, Politecnico Di Milano, Via La Masa 1, 20156 Milan, Italy; 2 Berlin, Germany; 3 TECHNOLOGIC S.R.L. Hologic Italia, Lungo Dora Voghera, 34/36A, 10153 Turin, Italy; 4 Nuclear Medicine-Bone Metabolic Unit, Fondazione IRCCS Cà Granda Ospedale Maggiore Policlinico, via Francesco Sforza 75, 20122 Milan, Italy

**Keywords:** Trabecular bone, Dual X-ray absorptiometry, Micro-computed tomography, 2D and 3D finite element models

## Abstract

The comprehension of trabecular bone damage processes could be a crucial hint for understanding how bone damage starts and propagates. Currently, different approaches to bone damage identification could be followed. Clinical approaches start from dual X-ray absorptiometry (DXA) technique that can evaluate bone mineral density (BMD), an indirect indicator of fracture risk. DXA is, in fact, a two-dimensional technology, and BMD alone is not able to predict the effective risk of fractures. First attempts in overcoming this issue have been performed with finite element (FE) methods, combined with the use of three-dimensional high-resolution micro-computed tomographic images. The purpose of this work is to evaluate damage initiation and propagation in trabecular vertebral porcine samples using 2D linear-elastic FE models from DXA images and 3D linear FE models from micro-CT images. Results show that computed values of strains with 2D and 3D approaches (e.g., the minimum principal strain) are of the same order of magnitude. 2D DXA-based models still remain a powerful tool for a preliminary screening of trabecular regions that are prone to fracture, while from 3D micro-CT-based models, it is possible to reach details that permit the localization of the most strained trabecula.

**Graphical abstract:**

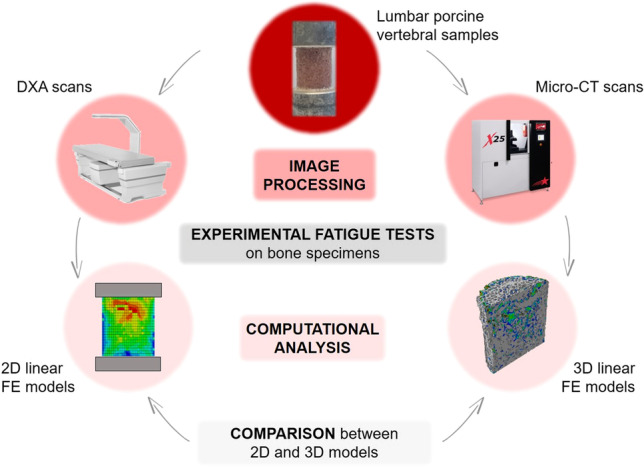

## Introduction

Bone fractures are an important source of morbidity and mortality in the elderly [1]. With the ageing process, human bone becomes more brittle and more prone to fracture [2] due to osteoporosis, a metabolic disease that reduces bone mineral density (BMD) and deteriorates bone microarchitecture leading to fracture [3].

A deep understanding of how fracture starts and propagates is then mandatory to develop effective preventive strategies. The initial step for fracture prevention is the understanding of bone mechanical properties. Bone is a complex, hierarchical composite material whose mechanical properties highly depend on the structural and material organization [4] and differ across hierarchical levels [5, 6]. The present work focuses on the investigation of bone damage processes in trabecular porcine vertebrae, by means of different approaches, depending on the considered resolution.

Clinical approaches start from dual X-ray absorptiometry (DXA) technique and measure areal bone mineral density (aBMD) and additional parameters such as the trabecular bone score (TBS), which quantifies trabecular texture, one of the various aspects of bone microarchitecture. However, although DXA is a widespread technique for low radiation, low cost, high reliability, and ease of use, BMD alone can predict only about 70% of vertebral fractures [7, 8].

With the support of finite element (FE) method, it is possible to obtain further insights related to damage initiation and propagation sites [9]. The power of computational tools is exploited by Naylor et al. [10] that implement FE models from baseline DXA scans of the hip to determine femoral bone strength and load-to-strength ratio, providing hints to improve bone strength assessment. Another recent attempt in FE modeling starting from DXA scans is performed by Colombo et al. [11]. In that study, they propose a strain-based parameter, named strain index of bone (SIB), to investigate bone resistance and support the diagnosis of vertebral fracture risk in clinical practice. The implementation of this novel numerical patient-specific approach is validated by means of static compressive mechanical tests on porcine vertebrae. The use of linear elastic FE models highly reduces the computational time, opening new opportunities to integrate the SIB with the currently used and accepted clinical fragility indexes [12, 13]. However, the anisotropy of trabecular structures, their spatial orientation and thickness, not captured by the DXA, are likely to play a crucial role in the determination of bone strength [14].

For this reason, high-resolution micro-computed tomography (micro-CT) is used in combination with more complex FE three-dimensional models for laboratory samples. Micro-CT permits the identification of bone microarchitecture in three dimensions, using X-rays to generate cross-sections of the bone. From the 3D image reconstruction, it is possible to obtain the fraction of a given volume of interest that is occupied by mineralized bone (bone volume, BV) with respect to the total bone volume (total volume, TV). The parameter BV/TV, usually reported as a % value, can give useful insights about bone damage. A preliminary micro-CT-based 3D FE model is performed by Müller et al. [15]: they implement a linear three-dimensional FE stress analysis, by testing under compression a sub-volume of trabecular bone from a quantitative CT scan. The meshing procedure with tetrahedrons requires very low computational time, but the considered volume is particularly small. A detailed definition of a damage criterion is performed by Hambli [16]. The author identifies a strain-based damage variable, focusing on the evidence that the failure process of bone is controlled by strain [17–25]. Additional studies [26] suggest that the accuracy of fracture risk assessment from BMD measured via DXA may be improved using a validated micro-CT-based FE analysis.

This paper is based on previous works [27, 28] that explore in detail both the experimental procedure to obtain trabecular cylindrical specimens starting from porcine vertebrae and the fatigue tests performed on the samples. Starting from the preliminary computational investigation with the strain-based criterion proposed in [11], the present work has the aim to provide deeper insights into the evaluation of damage initiation and propagation in trabecular vertebral samples by means of a comparison between DXA and micro-CT-based FE models.

## Materials and methods

**Fig. 1:**
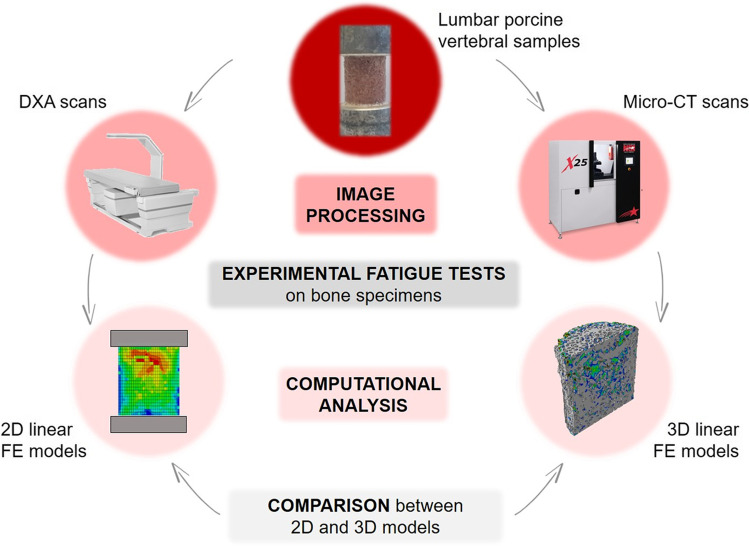
General overview of the work

The experimental and computational procedure of the present work is schematized in Fig. 1.

### Sample preparation

Twenty-seven cylindrical trabecular samples were cored from 6 lumbar vertebrae from 12- to 18-month-old animals, collected from a local butcher. Samples’ nominal diameter and height were 14 mm and 22 mm, respectively. The ends of the samples were glued into aluminum endcaps and the distance between those endcaps was 16 mm (effective length (*L*_eff_) of the sample).

The detailed procedure for sample size selection and sample preparation is described in [28].

### DXA scanning

DXA scans were performed by means of *Hologic Discovery*, *a system by Hologic, Inc., Marlborough, MA, USA*. The device was installed at the Bone Metabolic Unit of the Nuclear Medicine of the Fondazione IRCCS Ca’ Granda-Ospedale Maggiore Policlinico, Milan, Italy [28]. Hologic densitometers were dual-energy systems with pulsed kV between 100 and 140 kV. Image segmentation was performed automatically by S.W. Apex 3.3 installed on Hologic Discovery™.

BMD was measured through the APEX software, and TBS was automatically calculated with the software provided by the Medimaps Group. These parameters were derived by excluding the aluminum endcaps from the analysis. DXA scans were performed on the undamaged samples, at the end of each interrupted fatigue step (if planned) and after the mechanical fatigue test.

### Micro-CT imaging

Micro-CT images were collected, similarly to DXA scans, at each interrupted fatigue test and before and after the mechanical test. The used device was an *X-ray metrology CT system (X25, North Star Imaging Inc., Buckinghamshire, UK)* available at the Department of Mechanical Engineering, Politecnico di Milano, Italy. The voxel size was 25.6 × 25.6 × 25.6 μm; the scanning parameters were 60 kV and 150 μA. During the scan, specimens were submerged in a saline solution (NaCl 0.9%). Seven hundred projections were recorded, and image reconstruction was performed with the *X-View CT software*. Images’ post-processing consisted of different steps:

1. Filtration and binarization of the images by means of ImageJ software [29], with the additional use of the BoneJ plugin [30]:
  - Application of a Gaussian blur filter for images’ noise attenuation (standard deviation of the Gaussian distribution = 1.5). This filtration technique is commonly used in medical imaging [31].
  - Conversion to gray-level 8-bit images with Otsu local thresholding [32], obtaining segmented images. Otsu’s thresholding technique is a method based on searching for the threshold that minimizes the intra-class variance, defined as a weighted sum of variances of the background and foreground classes [33].
  - Isosurface extraction with the marching cubes algorithm [34].
  - Resampling of 2-in. pixel units to obtain the*.stl* file.
2. Reference system definition and additional noise removal using Rhinoceros software (developers Robert McNeel & Associates)
3. Mesh generation through HyperWorks™ (Altair Engineering, Inc.) software: 3D mesh generation with the shrink-wrapping technique [35, 36].

The final output was a 3D mesh with hexahedral elements, generated from the recommendations of [37], i.e., aspect ratio from 1 to 3, angle idealization < 160°, and Jacobian > 0.7. The choice of the element size and additional observations related to the mesh are reported in Sect. 2.4.2.

Micro-CT imaging was performed on the undamaged samples, at the end of each interrupted fatigue step (if planned) and after the mechanical fatigue test.

### Mechanical testing

High-cycle fatigue (HCF) testing was performed under force control at the frequency of 2 Hz and with a load ratio between the minimum (*F*min) and the maximum (*F*max) force *R* = *F*_*min*_*/F*_*max*_ = *0.1* [28]. During the tests, the specimens were submerged in saline solution (NaCl 0.9%).

The first set of fatigue tests (21 samples) was stopped either when the maximum displacement reached 2 mm or when the cycles exceeded 8·10^5^, i.e., runout condition [28, 38–40]. The second set of 6 samples was loaded with interrupted fatigue tests.

### Computational 2D and 3D models

2D and 3D computational models were implemented with the aim to analyze damage evolution in trabecular bone samples. Both the numerical approaches of the present study were based on the FE method and were implemented with the commercial software ABAQUS (v. 2017, SimuliaTM, Dassault Systèmes®).

They consisted in (1) a 2D modeling strategy based on DXA scans and (2) a more complex and detailed 3D approach, based on micro-CT. All the models were linear elastic.

Different numerical models were developed by considering the DXA and micro-CT scans before and after the experimental tests in order to study the damage accumulation. FE analyses are powerful tools to predict the zones more prone to incipient damage and how the damage propagates. The strain field was the quantity selected for incipient damage identification. In particular, the attention was focused on the region where the highest strain, considered in absolute value, occurred. Indeed, being the simulated test under compressive load, the minimum principal strain was the monitored field quantity both for 2D and 3D models.

### 2D DXA-based FE models

The DXA output was a map of squared pixels in grayscale, obtained from the bone density, with a resolution of 500 × 500 μm (i.e., 500 μm/pixel). This was also the size of the squared FEs (plane strain, type CPE4 in ABAQUS).

The thickness associated with each FE model was constant and derived from the equivalence between the cross-section of the real cylindrical specimen and the cross-section of the 2D-FE model. Material behavior was linear elastic. The elastic modulus of each FE analysis was calculated from the global elastic modulus *E*_*0*_, experimentally measured at the beginning of the compressive fatigue test on each specimen, as:

1 $${E}_{0}=\frac{F/{\mathrm{min}(A}_{eff})}{u/{L}_{eff}}$$

where *F* was the loading force; ${A}_{eff}$ the specimen-specific effective bone area measured via micro-CT and calculated as:

2 $${A}_{eff,i}={A}_{n} \cdot {\left(BV/ TV\right)}_{i}$$

where *A*_*n*_ was the nominal area of the sample, *i* was the i-th slice of the sample, and *u* was the crosshead displacement. This global *E*_*0*_ value was then associated with each finite element, based on the gray level of the DXA image. In other words, the grayscale map generated a corresponding map of local elastic moduli.

Two conditions applied during the analyses simulated the uniaxial tests: (1) clamp at the bottom edge nodes; (2) application of a vertical compressive force *F*, uniformly distributed to the upper edge nodes. The value of *F* was the maximum compressive load applied during the experimental tests. The three experimental maximum force levels applied during fatigue tests were 800, 1000, and 1200 N. 2D numerical models are developed starting from the DXA obtained by all the samples subjected to fatigue tests. Numerical results consisted of the minimum principal strain field ε_p,min_, post-processed to estimate the SIB, defined as (Colombo et al., 2019):

3 $$SIB=max\left|{\varepsilon }_{p,min}\bullet {10}^{4}\right|$$

It is worth mentioning that, within the finite element framework, strains were estimated at the 4 integration points of each element.

One value of SIB that corresponded to the most strained point of the specimen was associated with each sample subjected to fatigue test.

In addition, a specific sample, called SP4, was considered for more detailed analyses. SP4 was subjected to a force amplitude *F*_*a*_ = 540 N, (*F*_*max*_ = 1200 N, *F*_*min*_ = 120 N), and the fatigue test was interrupted after 1000, 2000, and 10,878 cycles (at 10,878 cycles the specimen failed due to crashing). For this sample, the SIB values of the 4 integration points were averaged over each element; then, the maximum value for each row of elements was estimated. In other words, this operation allowed calculating a value of SIB for each cross-section of the specimen. Plotting the SIB trend as a function of the height would allow recognizing the weakest cross-section of SP4, i.e., the region characterized by the highest SIB values. To identify damage evolution during interrupted fatigue testing of this specimen, four DXA-based FE models of the sample were implemented in different damage conditions: (1) pre-fatigue, (2) first interruption after 1000 cycles, (3) second interruption after 2000 cycles, and (4) post-fatigue after 10,878 cycles. Table 1 reported the values of the elastic modulus experimentally measured at each interruption that were used as input for the FE simulations.

**Table 1:** Experimental values of the elastic modulus, measured at each cyclic test interruption

Cycles [-]	*N* = 0 (Pre-fatigue)	*N* = 1000 (interrupted_1)	*N* = 2000 (interrupted_2)	*N* = 10,878 (Post-fatigue)
E [MPa]	3029	2817	2575	1727

### 3D micro-CT-based FE models

**Fig. 2:**
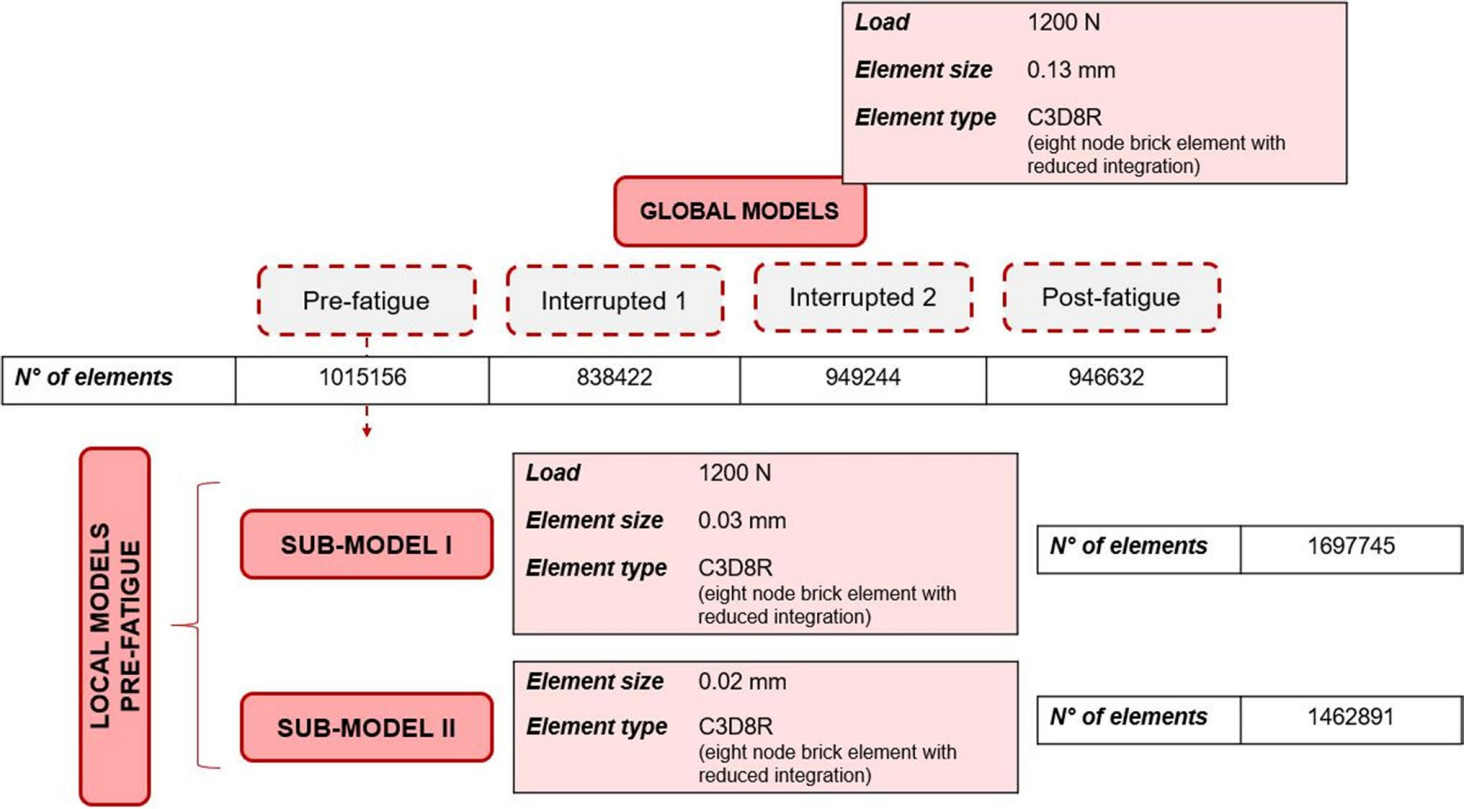
Overview of the implemented 3D micro-CT-based models. For the four global models and the two sub-models, the applied load, the element size and element type of the mesh are reported

2D DXA-based models could be potentially implemented in clinical routine, but they had also some limitations, due to DXA low resolution and to the simplification with a 2D geometry. Laboratory micro-CT images offered the chance to implement a more refined 3D simulation of the previously defined sample. The numerical procedure to study damage evolution was similar; four 3D FE models of the whole sample, named global models, were implemented, corresponding to the 4 damage conditions of the interrupted cyclic test. The characteristics of the global models in terms of element size, number of elements, element type, and applied load are reported in Fig. 2.

Two additional sub-models of the undamaged (pre-fatigue) sample were then implemented with the crucial aim to obtain a precise localization of the failure process’s origin. In addition to this, the sub-models allowed overcoming the limitations in mesh refinement encountered with the global model, by reducing the dimension of the volume of interest and the element size too. These sub-models focused respectively (1) on the weakest cross-section (or failure band) of the specimen’s global model, for the *sub-model I,* and (2) on the weakest trabecula of this failure band, for the *sub-model II*. The initial identification of the failure band and the following definition of the most strained trabecula were possible by implementing an increased mesh refinement process, as reported in Fig. 2.

An overview of the implemented 3D models is reported in Fig. 2.

Uniform and constant material properties were set to the bone material. Poisson ratio was set to 0.3, while Young modulus was estimated from the experimental force and displacement values at the beginning of the test on the selected specimen: *E*_o_ = 3029 MPa (Table 1).

Hexahedral mesh (linear brick elements, type C3D8R in ABAQUS) was chosen over tetrahedral mesh since it was highly recommended for models that involve bone tissue. A convergence analysis was performed for the choice of a suitable element size for the global model and the two sub-models. A compressive force *F* = 1200 N was applied at the top surface of the global models and *sub-model I*: *F* corresponded to the maximum applied load for the considered specimen. The bottom surface was clamped to simulate the boundary condition given by the lower endcap. The displacement field of the *sub-model I* was then used as boundary condition for the *sub-model II* of the pre-fatigue condition.

Numerical 3D results consisted in the minimum principal strain field, similarly to the SIB for the 2D analysis. It must be taken into account that locally bone fracture was due to trabecular instability when a trabecula was subjected to a compressive load [20, 41].

## Results

For this work, the computational results are presented in terms of 2D and 3D outcomes. The results obtained in terms of experimental tests are already presented in detail in [42].

### 2D DXA-based results

**Fig. 3:**
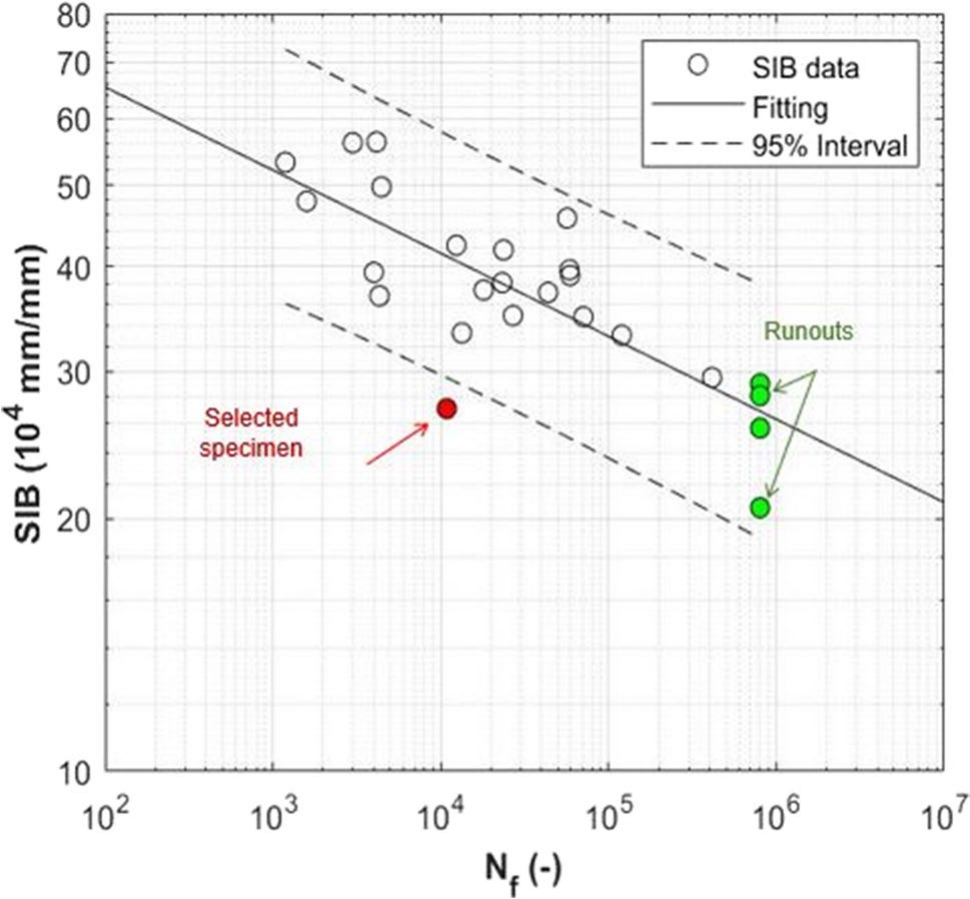
Log–log plot of SIB as a function of the number of cycles to failure *N*_f_. In red it is evidenced the specimen SP4 selected for further 3D numerical analyses; specimens in green are the runouts (not failed after 8∙10^5^ cycles). The least-square regression, solid line, is based on the law: $SIB=a {N}_{f}^{b}$. The upper and lower symmetric prediction bounds, dashed lines, are evaluated with the T-student distribution with 95% probability

Figure 3 shows the results of the 2D DXA-based simulations in terms of SIB, calculated on undamaged samples, as a function of the number of cycles to failure *N*_*f*_. Data of all the available specimens from [28] are fitted with a power least-squares interpolation based on the Wӧhler model, that results linear in the log–log plot:

4 $$SIB=a {N}_{f}^{b}$$

where the two coefficients are *a* = 0.796∙10^4^ mm/mm and *b* = 2.013. The determination coefficient *R*^*2*^ of this interpolation is 62.5%, and the adjusted *R*^*2*^_*adj*_ is 60.9%, with *p* value < 0.001. In Fig. 3, also 95% prediction bounds are added. The specimen highlighted in red, and called SP4, is selected for further numerical investigation.

**Fig. 4:**
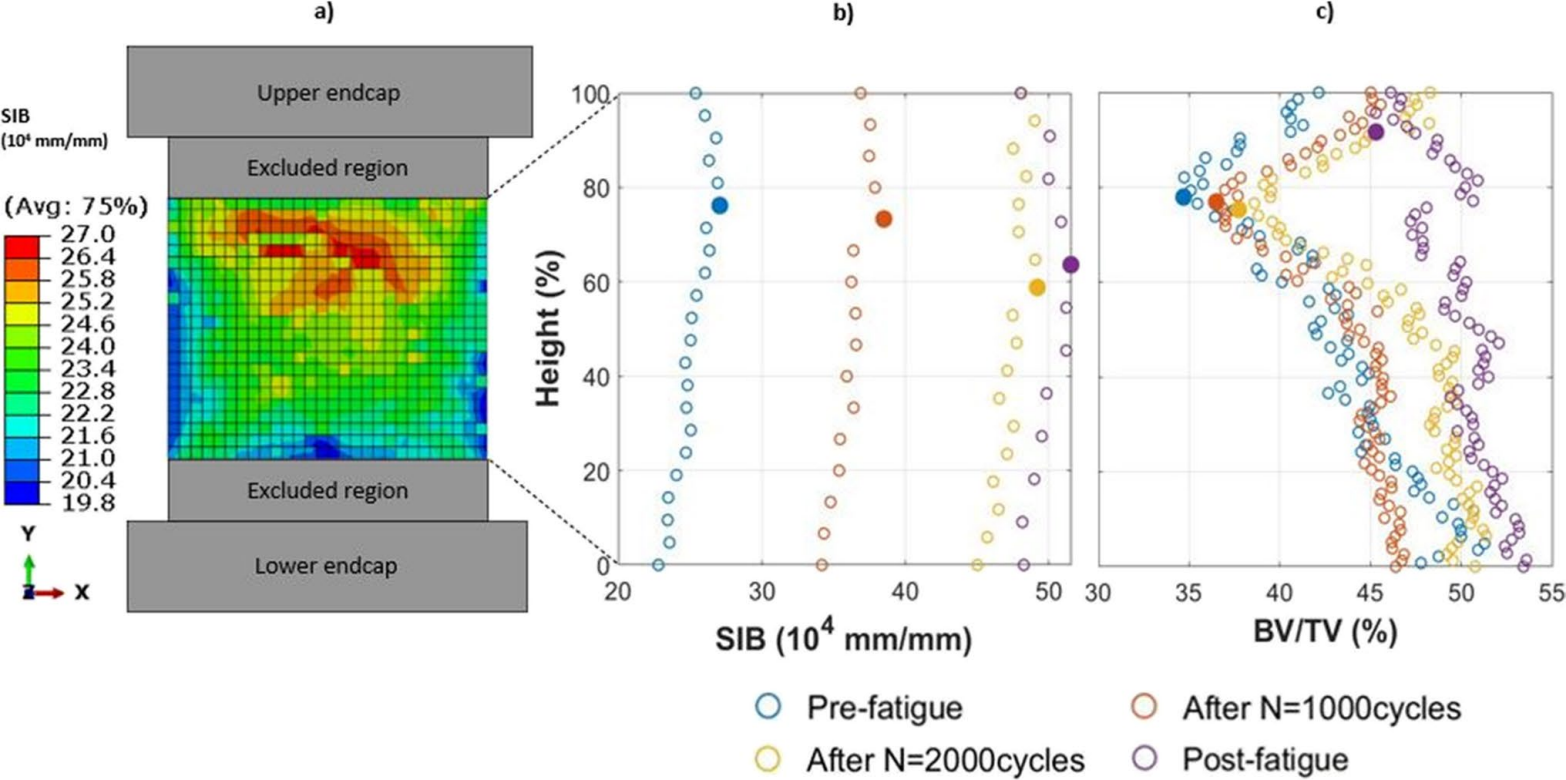
Results of the selected specimen: **a** SIB field from the 2D DXA-based numerical model; **b** values of SIB as a function of the normalized specimen’s height; **c** values of BV/TV from µ-CT scanning as a function of the normalized specimen’s height. Filled circles correspond to (**b**) the maximum SIB and (**c**) the minimum BV/TV. BV/TV was calculated as an average of every eight stacks of images from micro-CT images

Figure 4a shows the SIB field for the identified specimen; the plots of Fig. 4b and Fig. 4c show the trend of SIB and BV/TV, respectively, as a function of the specimen’s height. SIB data are averaged per row, while BV/TV are calculated as an average of every eight stacks of images from micro-CT images, as for the specimens processed in [28]. This comparison between the SIB and the BV/TV values will be particularly useful in order to localize the most strained regions identified by 2D FE models with respect to the 3D architectural variations of the sample.

### 3D micro-CT-based results

3D numerical models based on micro-CT images are developed on the sample SP4 previously defined and reported in red in the diagram of Fig. 3.

**Fig. 5:**
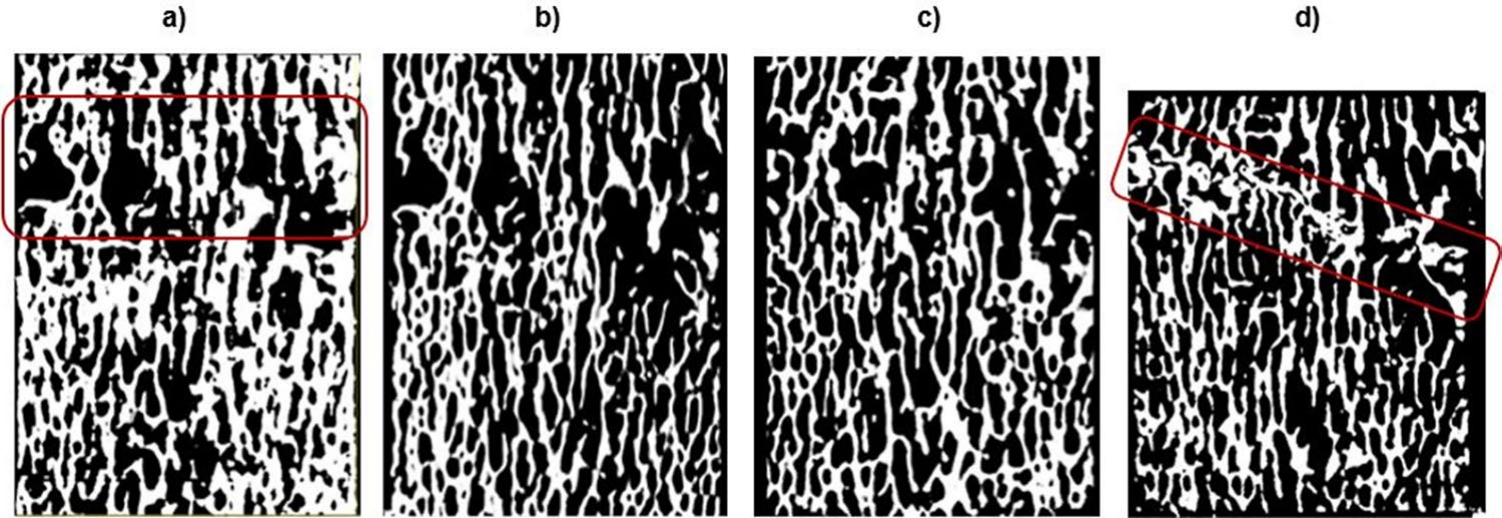
Binary images of the axial section of the selected specimen: **a** pre-fatigue; **b** after 1000 cycles; **c** after 2000 cycles; **d** at the end of the fatigue loading, *N*_f_ = 10,878 cycles

Figure 5 shows the binary images of the cross-section of the selected sample at undamaged condition (pre-fatigue), after 1000 loading cycles (interrupted_1), after 2000 loading cycles (interrupted_2), and at the end of the fatigue test (post-fatigue).

All the numerical results of the global models (pre-fatigue, interrupted_1, interrupted_2, and post-fatigue) are reported in terms of the minimum principal strain.

**Fig. 6:**
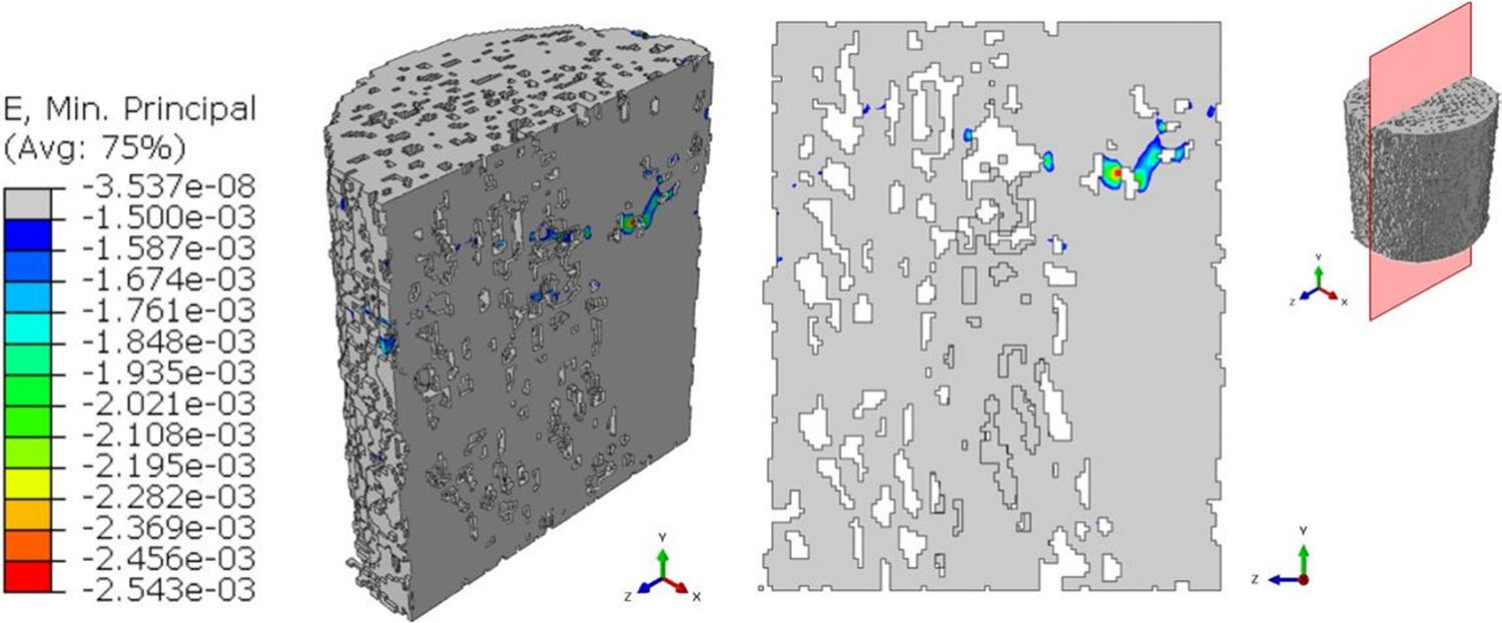
Output of the global pre-fatigue model. Contours of the minimum principal strains are presented in a 3D view. The slice with the maximum (in absolute value) ε_p,min_ is reported

Figure 6 shows the results obtained for the pre-fatigue model: the peak value of the minimum principal strain is equal to 2.543∙10^–3^. The figure indicates the localization of the peak strain in the three-dimensional view and the corresponding 2D section. The chosen surface resampling’s threshold of 2 is a compromise between the need for a precise meshing over the pixel grid and the necessity to reduce the heaviness of the mesh and the consequent computational time.

**Fig. 7:**
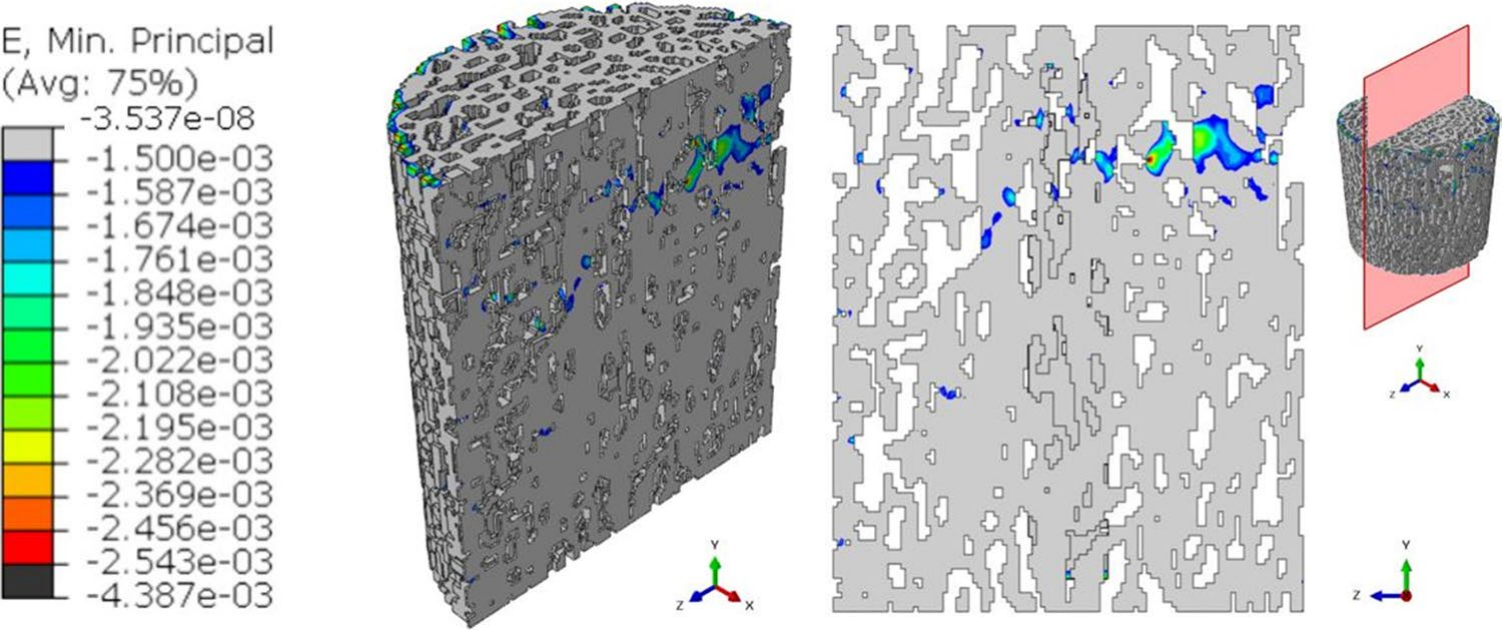
Output of the global interrupted_1 model. Contours of the minimum principal strains are presented in a 3D view. The slice with the maximum (in absolute value) ε_p,min_ is reported. In the legend, the black color represents ε_p,min_ values that overcome the maximum ε_p,min_ (in absolute value) reached by the pre-fatigue model

The maximum value of the minimum principal strain increases for the interrupted_1 model (Fig. 7), reaching a peak of 4.387∙10^–3^ in absolute value.

**Fig. 8:**
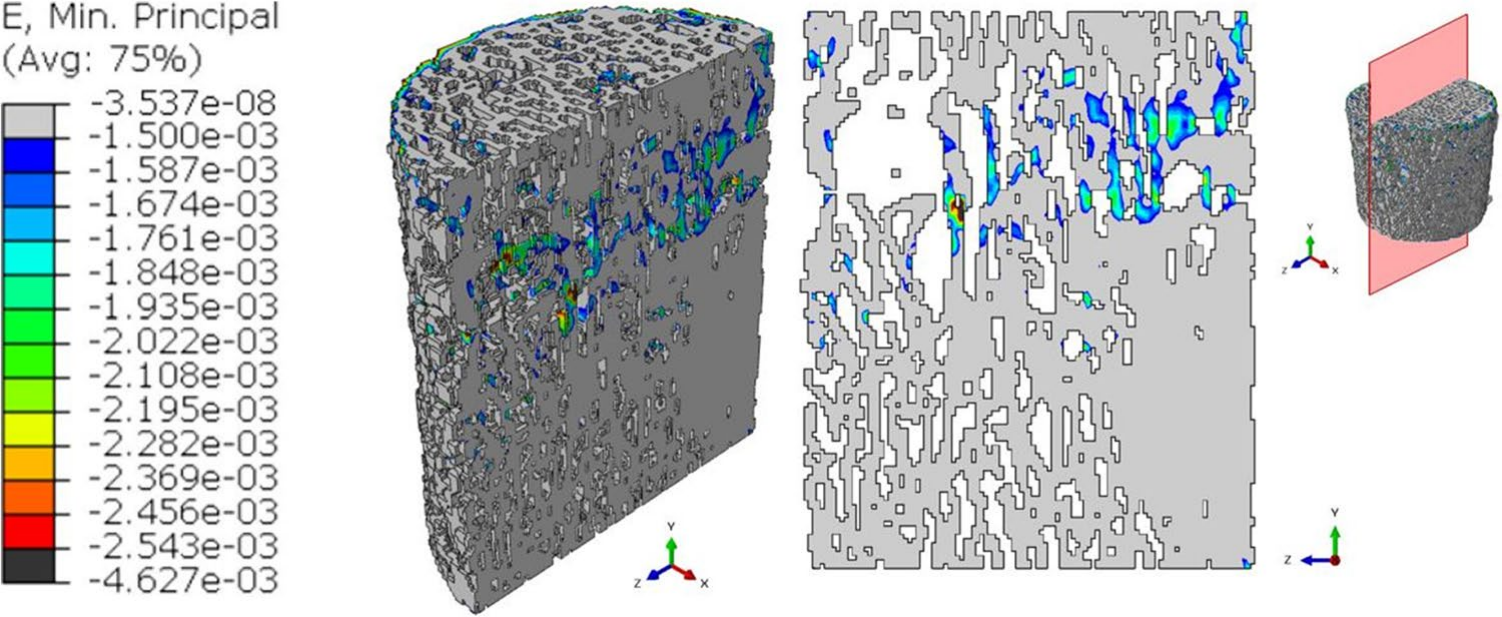
Output of the global interrupted_2 model. Contours of the minimum principal strains are presented in a 3D view. The slice with the maximum (in absolute value) ε_p,min_ is reported. In the legend, the black color represents ε_p,min_ values that overcome the maximum ε_p,min_ (in absolute value) reached by the pre-fatigue model

The same increasing trend in the strained regions is visible for the interrupted_2 model (Fig. 8).

**Fig. 9:**
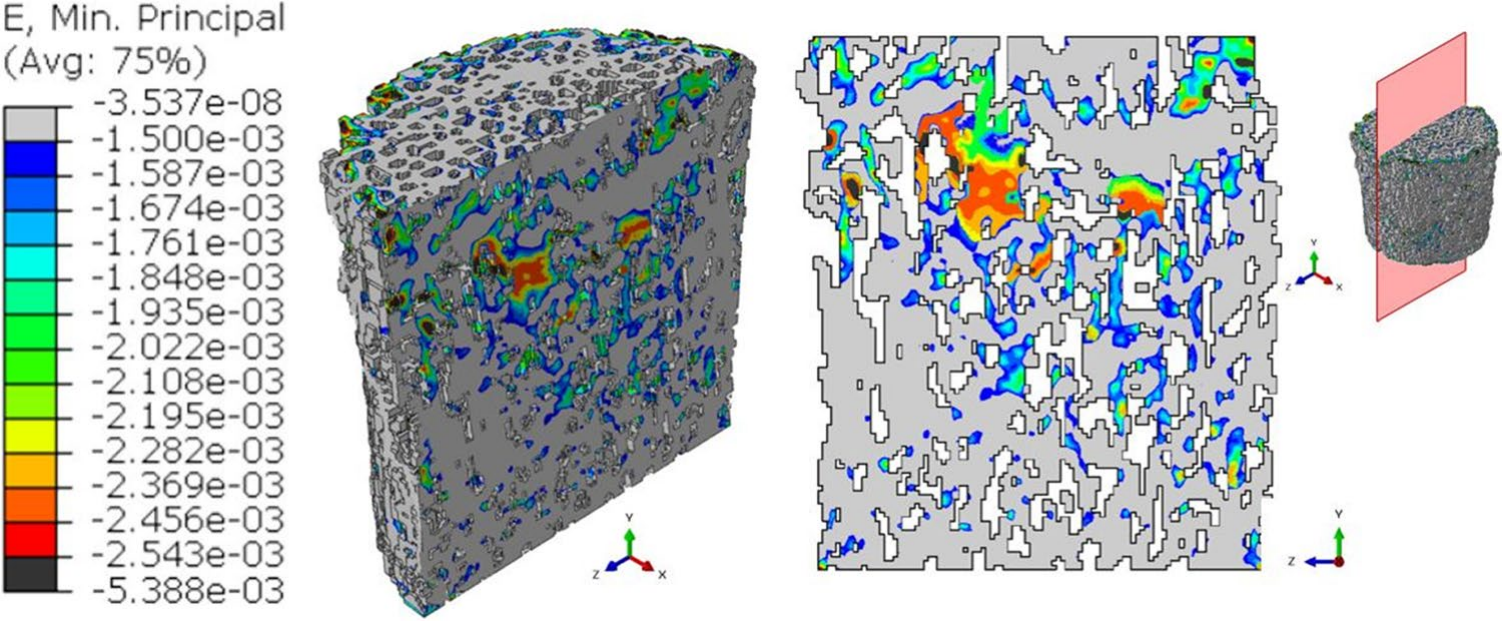
Output of the global post-fatigue model. Contours of the minimum principal strains are presented in a 3D view. The slice with the maximum (in absolute value) ε_p,min_ is reported. In the legend, the black color represents ε_p,min_ values that overcome the maximum ε_p,min_ (in absolute value) reached by the pre-fatigue model

The structure is collapsed in the post-fatigue simulation, where the most strained regions in the yz plane reach 5.388∙10^–3^ (Fig. 9).

**Fig. 10:**
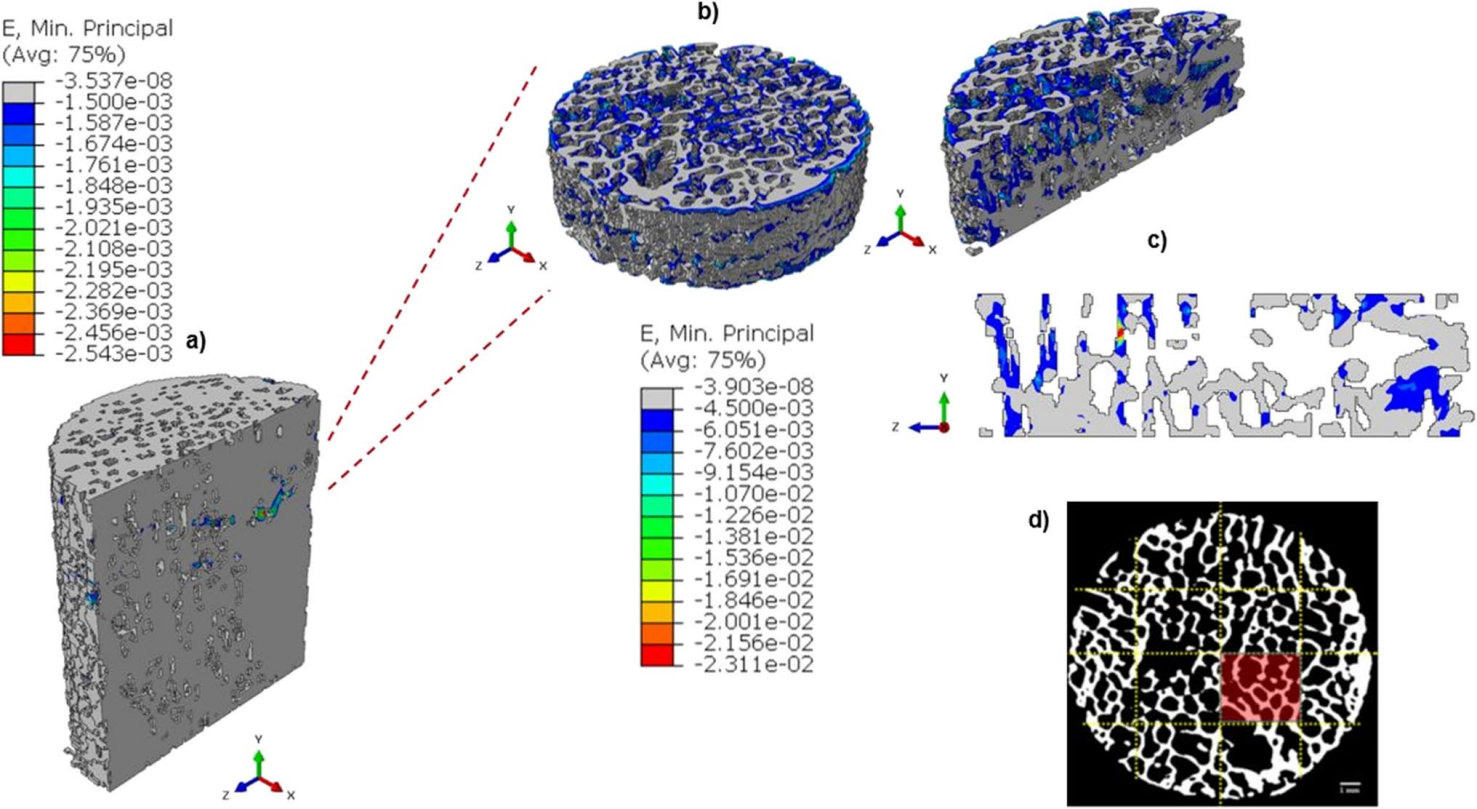
Results of the *sub-model I* in terms of ε_p,min_. From the global pre-fatigue model (**a**), it is selected a 4-mm-height disk (**b**). The most strained slice and its three-dimensional view are reported in (**c**). In (**d**) this critical region is localized in the micro-CT slice of the SP4 sample in pre-fatigue condition

As regards local models, the *sub-model I* takes into account a 4 mm thick disk from the pre-fatigue global model (Fig. 10).

**Fig. 11:**
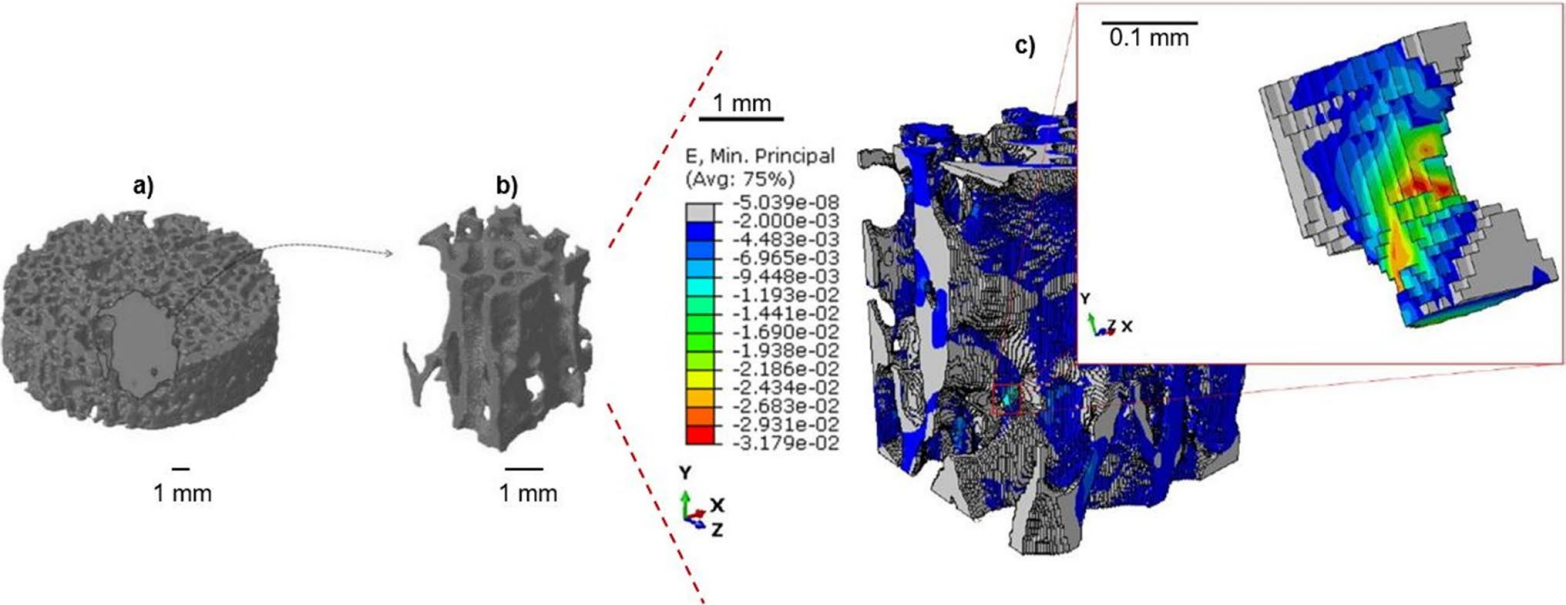
From the *sub-model I* (**a**), the most strained zone is identified (**b**). In (**c**) the single trabecula with the maximum | ε_p,min_| is reported

In *sub-model I*, the highest (in absolute value) minimum principal strains are highly localized. Hence, a portion of a brick shape (3 × 3 × 4 mm) is extracted, generating the *sub-model II*, that allows localizing the most strained trabecula (Fig. 11). In both sub-model I and sub-model II, the minimum principal strain values in absolute value (10^–2^) are one order of magnitude higher with respect to the global model (10^–3^). This implies that the performed mesh refinement in the sub-models permits to capture more in detail the trabecular network, identifying thinner trabeculae that are not visible in the global model and that are more prone to fracture.

## Discussion

The results of the 2D and 3D numerical models shown in the previous section are given in terms of minimum principal strains, following some literature evidence that fracture in bones is associated with a strain-based criterion, e.g., [21].

In the current research, 2D numerical models based on DXA scans are implemented and analyzed plotting the SIB parameter, introduced in Colombo et al. (2019). In that paper, the 2D numerical approach was validated, based on a comparison between the experimental and numerical elastic modulus. On the other hand, the 3D numerical models are validated by comparing the experimental and numerical stiffness of the undamaged sample. These values are respectively equal to 5219 N/mm and 4367 N/mm, i.e., the percentage difference is 16%.

Hence, given the validity of the numerical models and of the obtained results, some comments for 2D and 3D models and a comparison between them can be drawn.

Focusing on DXA-based results (Fig. 3), it is possible to underline that SIB data as a function of fatigue life *N*_*f*_ experience a good linear fitting: these outcomes seem suitable to account for the progressive damage in trabecular bones.

Among the specimens in Fig. 3, the attention is focused on the sample SP4; see Fig. 4. Figure 4a presents the SIB values over the undamaged sample, evidencing a weaker region, close to the top endcap. Figure 4b, c compares the numerical SIB with the experimental BV/TV as a function of the interrupted steps. The discussion can be done in parallel, from two viewpoints: (1) the trends of SIB and BV/TV as a function of the applied number of cycles and the progressive damage and (2) the region where they show criticality, i.e., the maximum SIB and the minimum BV/TV.

SIB (Fig. 4b) shows an average tendency to increase with respect to the number of cycles for the considered sample. The relative percentage differences in SIB values with respect to the pre-fatigue are: ΔSIB_Interrupted_1-Pre-fatigue_ = 42%; ΔSIB_Interrupted_2-Pre-fatigue_ = 82%; and ΔSIB_Post-fatigue-Pre-fatigue_ = 91%. On the contrary, the relative increase in the SIB trend at the different fatigue interruptions become smaller (ΔSIB_pre-fatigue-Interrupted_1_ = 42%; ΔSIB_Interrupted_1-Interrupted_2_ = 40%; ΔSIB_Interrupted_2-Post-fatigue_ = 9%).

We can hypothesize that the specimen is highly damaged already after 2000 cycles. After the interrupted_2, the trabecular structure starts collapsing, causing a change in the trabecular morphology. Indeed, BV/TV trends (Fig. 4c) support this hypothesis, showing curves almost overlapped up to 2000 cycles, while at the end of the fatigue life BV/TV experiences higher values. In particular, the weakest region, initially with less material, becomes as dense as the rest of the specimen due to the collapse of trabeculae.

On the other hand, considering these trends as a function of the specimen’s height, other considerations can be drawn. In correspondence of the highest SIB values for both the pre-fatigue and the interrupted_1 condition, it is possible to find the lowest value of BV/TV (filled circles in Figs. 4b, c); i.e., less bone volume is present. This region with the highest strains is located at about 70–80% of the specimen’s height. Instead, when the specimen reaches 2000 cycles, the SIB parameter highlights another section (at 60% of the total height) as the most strained one, while BV/TV still underlines the previous one at 75% of the height as the most critical. This could mean that the region with less material is almost failed and prone to collapse. Hence, it is likely that it cannot withstand further load: strain concentration is shifted towards other regions that are still able to resist compression. At the end of the test, indeed, the most strained region, i.e., the one with the highest SIB, remains continuously positioned at about 60% of the height, while BV/TV evidences the crushing with an almost constant trend over the height. Eventually, the region with the lowest BV/TV is at 92% of the height. From these findings, it could be observed that, increasing the number of fatigue cycles, trabeculae progressively break, leading to a reduction in the BV/TV values. This happens before the post-fatigue condition, in which the specimen is fully collapsed and the BV/TV increases in presence of a reduced TV.

It is worth noting that this discussion is also supported by the micro-CT images of the specimen in Fig. 5. These are all axial sections of the specimen and they allow for better visualization of the change in the inner bone structure. At the beginning of the test (Fig. 5a), it is clearly visible a less dense region, which is the same identified as the weakest by the 2D simulation (Fig. 4a). Similar considerations are valid for the micro-CT scans at the interrupted_1 and interrupted_2 (Fig. 5b and c). The final configuration, at the end of the fatigue test (Fig. 5d), evidences that the trabeculae belonging to this region are collapsed, and a compact geometry is generated. The sample height is reduced by 2 mm, that is, the failure condition mentioned in Sect. 2.3. This damaged region is slightly inclined, and damage follows a preferential plane, depending on the original trabecular spacings’ distribution of the bone tissue.

Deeper insights can be found when analyzing the outputs of the 3D models in terms of the strain values occurring in the specimen. Comparing the minimum principal strain field of Figs. 6, 7, 8, and 9, it is visible a progressive increase in the maximum value (in absolute terms) of the minimum principal strain. In addition to this, an evident increase in the most strained regions is detectable from the four global models. At the beginning of the test, peak strains are localized in correspondence of a limited region around a wide cavity of the specimen (Fig. 6). The position of this region corresponds to the one identified in the 2D analysis. After 1000 cycles, the same region undergoes higher strains (Δ(max|ε_p,min_|)_Interrupted1-Pre-fatigue_ = 73%). After 2000 cycles, the whole axial-section corresponding to this region is highly strained (Δmax|ε_p,min_|_Interrupted_2-Pre-fatigue_ = 82%), evidencing a failure band similar to other literature trabecular bone fracture modes [39, 43]. The peak strain changes its spatial location due to the damage propagation. At the end of the test, the damaged region is significantly larger with respect to the previous outcomes obtained in the interrupted_1 and interrupted_2 (Δmax|ε_p,min_|_Post-fatigue-Pre-fatigue_ = 112%). This underlines that the damage is widespread and that many trabeculae are already failed. The field of Fig. 9 reflects the change in the morphology occurring into the bone tissue, evidenced using micro-CT images in Fig. 5d.

In order to understand damage initiation sites, further analyses are run on the untested sample, isolating a cylindrical failure band at the region where peak strains occur (the *sub-model I* of Fig. 10). Within this model, a second sub-model, i.e., *sub-model II* of Fig. 11, helps in evidencing the most strained trabecula which is responsible for the damage initiation. Figure 11 shows this trabecula, which is approximately perpendicular to the y-axis, i.e., the direction of the compressive load. This is consistent with [44] that simulated an ovine trabecular sample with a complex 3D model, including damage progression. The trabecula experiences compressive strains: this can suggest that the damage mode of this bone portion is related to local instability, leading to bending and to the final collapse that is visible macroscopically in Fig. 5d and Fig. 9. This numerical procedure and the 3D FE results suggest that damage starts as a local microstructural phenomenon, due to bending tensile strain, before spreading to the whole complex trabecular structure.

Comparing 2D and 3D analyses, it is possible to underline that SIB peak in the 2D model (Fig. 4a) is located at about the same height where the peak of minimum principal strain occurs in the 3D model (Fig. 6), i.e., where the sub-modeling technique identifies the most strained trabecula (Fig. 11).

**Fig. 12:**
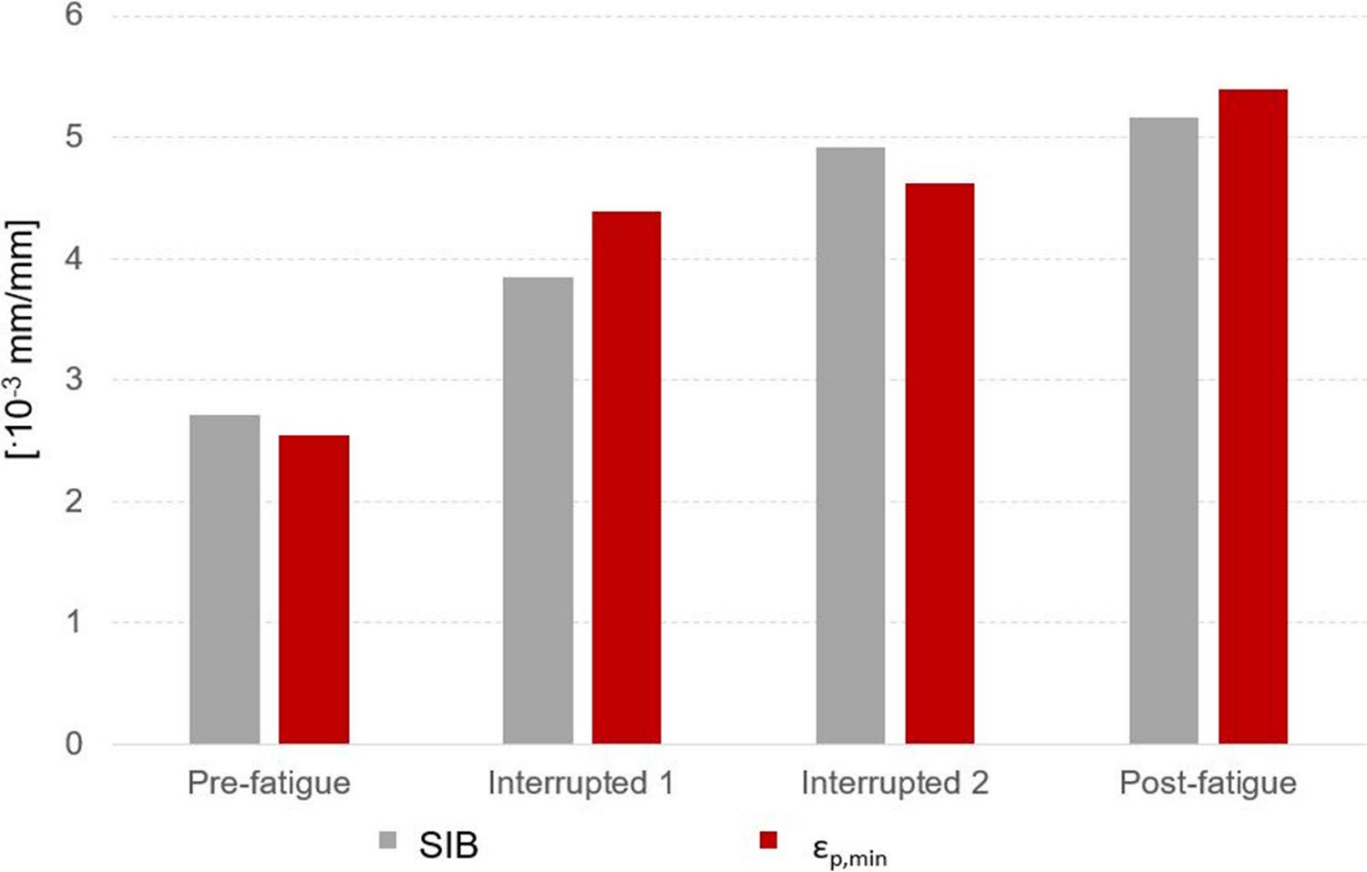
Summary of the SIB and of the minimum principal strain values obtained from the 2D and 3D models, respectively

Besides, values of strains estimated with the 2D approach (i.e., the SIB) and the 3D approach (i.e., the minimum principal strain), are quite similar (see Fig. 12), with percentage differences between − 12 and + 6%. In other words, more complex 3D analyses based on micro-CT images help in understanding the failure mechanism but also underline that the parameter SIB can catch local strain concentrations, despite all the limitations of a bi-dimensional model.

However, the power and the details of the 3D model are not achievable with the 2D simplification. In particular, the peak strain on the trabecula measured with the last sub-model is the highest and allows to better understand the premature failure of the specimen shown in Fig. 3. Indeed, the strain on the weakest trabecula, e.g., 3.2% (see Fig. 11), is within the order of magnitude of experimental literature values for the ultimate failure strain, obtained from compressive tests. It is possible to mention a few of them: 1.71 ± 0.49% for porcine vertebral trabecular specimens [27], 2.91% for ovine vertebral trabecular specimens [44], and 3.1% for bovine vertebral trabecular specimens [45]. This comparison underlines how the damage is a local phenomenon starting from weaker regions within these trabecular structures and spreading to the rest of the sample during further load applications.

However, some limitations may arise from this work. More in depth, the implemented 2D and 3D models are linear elastic and bone is considered as isotropic. In order to obtain more reliable outcomes, it must be considered that bone exhibits complex behavior including anisotropy, viscosity, and remodeling abilities. Nevertheless, the possibility to introduce bone nonlinearities in the mechanical behavior will lead to extra degrees of complexity in the analysis, increasing the already high computational effort too. The FE models implemented in the current work could be considered as a powerful preliminary screening in the localization of multiscale damage.

## Conclusions

The main outcomes of the work are summarized as follows:

- 2D DXA-based and 3D micro-CT-based linear elastic FE models are developed to localize damage initiation and propagation sites at different magnification levels. The models are validated using experimental fatigue testing on porcine lumbar vertebrae.
- A detailed comparison between the outputs of both models in terms of minimum principal strain is presented.
- Simplified 2D models can identify regions with higher strains, but they are not capable of providing further insights into bone damage processes. Conversely, 3D models show minimum principal strain contours at the single trabecula level.
- The capability of 3D models in localizing fracture sites is permitted by the implementation of sub-models with refined meshes with respect to the global model of the whole trabecular sample.
- 2D DXA-based models still remain a powerful tool for preliminary screening of trabecular regions that are prone to fracture and represent an interesting connection with the actual clinical approach. 3D micro-CT-based models represent a research approach that will help in the comprehension of damage progression.
